# Don’t Judge a Book by Its Cover: The Role of Statins in Liver Cancer

**DOI:** 10.3390/cancers15205100

**Published:** 2023-10-22

**Authors:** Natalia Piekuś-Słomka, Lavinia Patricia Mocan, Rezarta Shkreli, Cristiana Grapă, Kinga Denkiewicz, Oliwia Wesolowska, Miroslaw Kornek, Zeno Spârchez, Artur Słomka, Rareș Crăciun, Tudor Mocan

**Affiliations:** 1 Department of Inorganic and Analytical Chemistry, Nicolaus Copernicus University in Toruń, Ludwik Rydygier Collegium Medicum in Bydgoszcz, Jurasza 2, 85-089 Bydgoszcz, Poland; natalia.piekus@cm.umk.pl; 2 Department of Histology, “Iuliu Hațieganu” University of Medicine and Pharmacy, 400349 Cluj-Napoca, Romania; trica.lavinia@umfcluj.ro; 3 Department of Pharmacy, Faculty of Medical Sciences, Aldent University, 1001-1028 Tirana, Albania; rezarta.shkreli@ual.edu.al; 4 Department of Physiology, “Iuliu Hațieganu” University of Medicine and Pharmacy, 400006 Cluj-Napoca, Romania; grapa.cristiana.maria@elearn.umfcluj.ro; 5 Department of Pathophysiology, Nicolaus Copernicus University in Toruń, Ludwik Rydygier Collegium Medicum in Bydgoszcz, 85-094 Bydgoszcz, Poland; 301685@stud.umk.pl (K.D.); 301637@stud.umk.pl (O.W.); artur.slomka@cm.umk.pl (A.S.); 6 Department of Internal Medicine I, University Hospital Bonn of the Rheinische Friedrich-Wilhelms-University, 53127 Bonn, Germany; miroslawkornek@web.de; 7 3rd Medical Department, “Iuliu Hațieganu” University of Medicine and Pharmacy, 400162 Cluj-Napoca, Romania; zsparchez@elearn.umfcluj.ro; 8 Department of Gastroenterology, “Octavian Fodor” Institute for Gastroenterology and Hepatology, 400162 Cluj-Napoca, Romania; 9 UBBMed Department, Babeș-Bolyai University, 400349 Cluj-Napoca, Romania

**Keywords:** statins, liver cancer, cholesterol, 3-hydroxy-3-methylglutaryl-coenzyme A reductase

## Abstract

**Simple Summary:**

Statins are a group of remedies developed to treat lipid disorders associated with cardiovascular diseases. Thanks to their high efficiency, they are the primary line of therapy for hypercholesterolemia. However, recent experimental and clinical studies indicate the potential use of statins in treating liver cancer. In this review, we discuss these aspects, bringing the reader closer to the importance of statins as a pharmacological tool in cancer prevention and the therapeutic management of the liver and bile duct neoplasm.

**Abstract:**

Statins, which are inhibitors of 3-hydroxy-3-methyl-glutaryl-coenzyme A (HMG-CoA) reductase, are an effective pharmacological tool for lowering blood cholesterol levels. This property makes statins one of the most popular drugs used primarily to prevent cardiovascular diseases, where hyperlipidemia is a significant risk factor that increases mortality. Nevertheless, studies conducted mainly in the last decade have shown that statins might prevent and treat liver cancer, one of the leading causes of cancer-related mortality worldwide. This narrative review summarizes the scientific achievements to date regarding the role of statins in liver tumors. Molecular biology tools have revealed that cell growth and proliferation can be inhibited by statins, which further inhibit angiogenesis. Clinical studies, supported by meta-analysis, confirm that statins are highly effective in preventing and treating hepatocellular carcinoma and cholangiocarcinoma. However, this effect may depend on the statin’s type and dose, and more clinical trials are required to evaluate clinical effects. Moreover, their potential hepatotoxicity is a significant caveat for using statins in clinical practice. Nevertheless, this group of drugs, initially developed to prevent cardiovascular diseases, is now a key candidate in hepato-oncology patient management. The description of new drug-statin-like structures, e.g., with low toxicity to liver cells, may bring another clinically significant improvement to current cancer therapies.

## 1. Introduction

Despite their rare occurrence, primary liver cancers are characterized by high mortality and are the second most common cause of cancer-related death. The two most frequent primary liver malignancies, whose incidence is growing rapidly worldwide, are hepatocellular carcinoma (HCC) and intrahepatic cholangiocarcinoma (iCCA). Nearly 85–90% of cancer liver cases are HCC, derived from hepatocytes, while iCCA, arising from cholangiocytes, is much less common (10–15%) [1]. Most risk factors for both HCC and iCCA are associated with chronic liver damage accompanying various diseases such as infection with hepatitis B and C viruses, cirrhotic liver disease, non-alcoholic fatty liver disease, chronic liver damage, and fibrosis [2,3]. The consequence of the aforementioned reasons for the occurrence of the discussed neoplasms is the geographical and sex variability of their incidence [3].

In the case of iCCA, surgical resection is the best and only potentially curative treatment. On the other hand, for HCC, local ablation or liver transplantation can also be curable, apart from surgical resection. Unfortunately, only a small number of patients are diagnosed at an early stage of the disease, when tumor size and/or number and no major vascular invasion on imaging or metastasis allow the procedures to be performed [3,4,5]. Patients in the intermediate and advanced stages of primary liver cancers are subjected to systemic therapy that could increase survival. First-line treatment for unresectable iCCA is platinum-based chemotherapy combined with gemcitabine or, less frequently, capecitabine, but the prognosis remains poor. Different molecularly targeted therapies and immunotherapies alone or in combination with cytotoxic drugs have been proposed in the last few years as second- and third-line therapies for iCCA [6,7]. The standard of first-line systemic treatment of HCC is based on tyrosine kinase inhibitors (sorafenib, lenvatinib, or dobafenib) or monoclonal antibodies (atezolizumab combined with bevacizumab or tremelimumab combined with durvalumab). Various molecular-targeted drugs are also the second line of HCC therapy due to the limited role of traditional chemotherapy in the treatment of advanced HCC [8]. Increasingly, chemo- or radio-embolization are also used to treat primary liver cancers. Unfortunately, these are also palliative methods aimed at extending survival and/or improving the quality of life [8,9,10].

Under the general term cholangiocarcinoma (CCA), apart from iCCA classified as primary liver cancer, there is also extrahepatic cholangiocarcinoma (eCCA), located within the hepatoduodenal ligament. Extrahepatic CCA can be divided into perihilar (pCCA) and distal (dCCA) diseases. The first is located between the cystic duct insertion and the second-order bile ducts, and the second originates in the common bile duct [11]. Of all CCA cases, 60–70% of cases are classified as pCCA, 20–30% are dCCA, and 5–10% are iCAA. While there has not been a significant increase in the incidence of eCCA (as for iCCA), the prognosis remains poor regardless of location [12,13].

The increasing incidence and low effectiveness of current treatment strategies make it necessary to search for new therapies that could be used as chemopreventive and treatment methods for primary liver cancers. Moreover, already-known drugs with a recognized safety profile that can be repurposed in this desired manner are sought [14,15].

The importance of statin drugs is evidenced not only by the number of scientific publications on the subject but, above all, by the fact that they are among the most frequently prescribed drugs globally [16]. More than 40 years have passed since the commercialization of the first statin—lovastatin, of natural origin [17]. Currently, seven statin drugs are approved by the Food and Drug Administration (FDA)—lovastatin, pitavastatin, atorvastatin, rosuvastatin, pravastatin, simvastatin, and fluvastatin. Cerivastatin was also available for several years but was withdrawn from the market due to serious side effects (primarily rhabdomyolysis), which were many times more likely to occur compared to other statins. Almost all statins are sold in the form of single-active ingredient tablets or capsules. Only simvastatin and atorvastatin are produced as fixed-dose combination drugs with amlodipine and ezetimibe [18].

The indication for statin prescriptions extends beyond treating dyslipidemia and cardiovascular disease. Functionally, all statins have a similar mechanism of action. The primary action mode of statins is the inhibition of 3-hydroxy-3-methylglutaryl coenzyme A (HMG-CoA) reductase (HMGCR), the microsomal enzyme involved in the biosynthesis of cholesterol (C_27_H_46_O). All statins bind to the active side of HMGCR and thus reversibly and competitively inhibit them. However, differences in chemical structure result in different potencies along with pharmacokinetic and pharmacodynamic properties [19]. Inhibition of cholesterol biosynthesis at the stage of mevalonic acid synthesis results in a decreased concentration of all downstream products. Thus, the use of statins affects the production of some crucial isoprenoid mediators, which are essential for activating different intracellular and signaling proteins that play important roles in multiple cellular mechanisms [20]. This is one of the explanations for the pleiotropic effect of statins, which is still the subject of studies referred to in numerous review papers [17,21,22].

This narrative review aimed to offer insight into the literature about statin drugs and their possible role in preventing and treating primary liver cancers. First, we discussed the properties and main use of currently available clinical practice statins in pharmacotherapies, then elucidated the molecular mechanism of their anti-cancer properties. Finally, we focused on the possible use of statins to prevent and treat HCC and CCA.

## 2. Statins—Chemical, Pharmacokinetic, and Pharmacodynamic Features—A Short Presentation

The beneficial relationship between improving the lipid profile and reducing cardiovascular risk has been known for many years. Several lipid-modifying drugs exist, i.e., fibrates, bile acid-binding resins, cholesterol-absorption inhibitors, and nicotinic acid. However, statins are the most widely used. They have been ordained since the late 20th century and nowadays are the most frequently prescribed drugs worldwide [16]. Currently, as mentioned earlier, seven statins (lovastatin, pitavastatin, atorvastatin, rosuvastatin, pravastatin, simvastatin, and fluvastatin) are approved by the FDA, and these drugs will be discussed.

**Figure 1:**
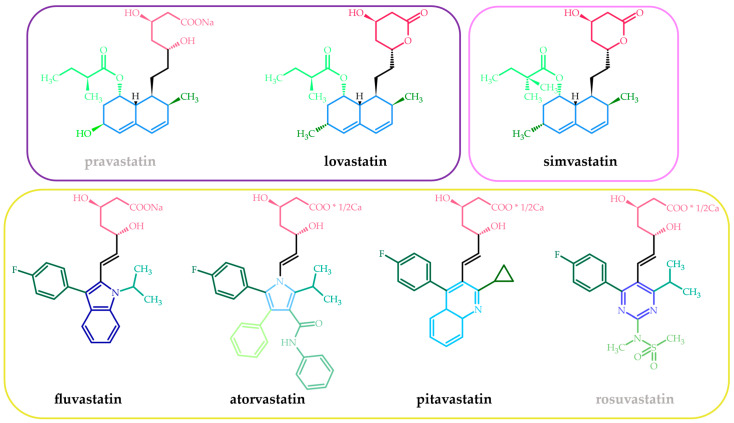
Chemical structures of FDA-approved statins. Different shades of red—different pharmacophores; different shades of blue—different ring structures; different shades of green—different side groups. The purple frame includes natural statins, the pink semi-synthetic, and the yellow synthetic. The gray text indicates hydrophilic statins.

Structurally, statins are a relatively homogeneous group of molecules. Three fragments, a pharmacophore, a ring structure, and side groups on the ring can be identified in their chemical structure (Figure 1) [23]. The pharmacophore of statins (the HMG-like moiety) is similar to the natural substrate of the HMGCR enzyme [24]. Moreover, the affinity of statins to the binding side of HMGCR is 8000 or higher than the endogenous substrate (HMG-CoA), which explains their high efficiency [25]. The HMG-like moiety can be present in an inactive lactone form (pro-drug) like in the natural origin lovastatin or semi-synthetic simvastatin (a simple modification of lovastatin consisting in the insertion into its side chain of an additional methyl group) or as a dihydroxyheptanoic acid unit in synthetic statins (atorvastatin, fluvastatin, pitavastatin, rosuvastatin) and pravastatin, which is of natural origin. The lactone moiety must first be hydrolyzed, mainly in the hepatocytes, to the acid form before binding to the active side of HMGCR [17]. The stereoselective binding process requires the statin to have a 3R, 5R configuration [26]. The ring structure and its side groups impart various degrees of lipophilicity, enabling maximum contact with the hydrophobic pocket of the HMGCR enzyme [24]. The aromatic fragments of statins are covalently linked to the pharmacophore and appear as partially reduced naphthalene along with a butyryl group in natural and semi-synthetic statins (lovastatin, pravastatin, and simvastatin), pyrrole (atorvastatin), pyrimidine (rosuvastatin), quinoline (pitavastatin), and indole (fluvastatin) [26]. The side groups (the butyryl group in statins with partially reduced naphthalene, the fluorophenyl group in the remaining ones, and possibly various others) on the ring define the solubility and, therefore, the pharmacological properties and pharmacokinetic characteristics of the statins. Therefore, rosuvastatin and pravastatin are considered hydrophilic (log D at pH 7.4 below 0 [27]), the polarity of which is increased by the methane sulphonamide group and the hydroxyl group, respectively [23,26].

As mentioned, two statins approved by the FDA are administered as lactone pro-drugs. The others are given as active compounds. The intestinal absorption of statins is quite variable but overall rapid. Peak plasma concentrations occur within four hours. The oral absorption range is 30–98%, but due to extensive first-pass liver metabolism, systemic bioavailability is down to 5–30% (except pitavastatin, with the highest bioavailability of approximately 80%). Food intake has a variable impact on statin absorption. When consumed with food, atorvastatin, fluvastatin, and pravastatin are less efficiently absorbed, whereas lovastatin has increased absorption with meals. No such effects are apparent for pitavastatin, simvastatin, and rosuvastatin. Hydrophilic statins require carrier-mediated uptake into the liver, while lipophilic statins can passively diffuse through the cell membrane in both hepatic and non-hepatic tissues. This results in greater hepatoselectivity of hydrophilic statins, which may reduce the incidence of adverse effects. As with other pharmacokinetic properties, statins’ metabolism depends on their lipophilicity. Lipophilic compounds undergo hepatic and enteric metabolism by the cytochrome P-450 enzymes. Both pitavastatin and rosuvastatin (water-soluble statins) undergo negligible metabolism via cytochrome P450. This explains why they do not participate in clinically relevant drug-drug interactions with CYP450 agents. After metabolism, lipophilic statins are predominately eliminated with the bile, whereas hydrophilic ones are eliminated essentially unchanged by both the kidney and liver. Most approved statins have short elimination half-lives of three hours or less. For this reason, they should be administered in the evening, when the rate of endogenous cholesterol synthesis is highest. This does not apply to atorvastatin, pitavastatin, and rosuvastatin, which have elimination half-lives greater than 10 h and can be taken any time of the day [20,22,23,25,26,27,28].

The primary mechanism of action of statins is based on inhibiting the HMGCR enzyme. As a result, the synthesis of non-sterol and sterol isoprenoids, particularly cholesterol, is repressed. Moreover, decreasing cholesterol concentration in hepatocytes causes up-regulation of low-density lipoprotein (LDL) receptor expression, which promotes the uptake of LDL and LDL precursors from the systemic circulation. The increase in plasma LDL clearance and the decrease in cholesterol synthesis are responsible for the statins’ cholesterol-lowering result [26]. The secondary mechanism of statins’ action is the inhibition of lipoprotein B100 synthesis in the hepatocytes and, consequently, the reduction in the synthesis and secretion of other triglyceride-rich lipoproteins [17].

The decreasing cholesterol biosynthesis determined by statins occurs at the proximal and rate-limiting steps of the mevalonate pathway (conversion of HMG-CoA to mevalonate). As a result of the inhibition of the mevalonate pathway, the synthesis of other essential products of this metabolic pathway, such as geranylgeranyl pyrophosphate (GGPP), farnesyl pyrophosphate (FPP), or isopentenyl pyrophosphate (IPP), is also inhibited. These intermediates are involved in the posttranslational modifications of numerous cell-signaling proteins, such as small monomeric GTPases (e.g., Ras, Rho, Rac, or Rap) and the γ-subunit of G-protein-coupled receptors, which importantly account for many biological processes. This is one explanation for the pleiotropic effect of statins. These pleiotropic effects are diverse in nature and include improving cardiovascular function, broad anti-inflammatory and anti-oxidant effects, enhancement of bone formation, anti-fibrotic effects, and reno-protective effects [17,20].

The chemopreventive and cancer-treating effects of statins, which are the subject of this paper, are discussed in numerous research studies conducted in vitro, in vivo, and both as population-based observational studies and interventional clinical trials [29,30,31,32,33]. Research into the effects of statins used both in monotherapy and in combination with other drugs was investigated, obtaining many positive results. These findings were highly dependent on statin type, dose, treatment period, and genetic and molecular characteristics of cancer. The beneficial anti-cancer effect is a result of both statins’ ability to inhibit the mevalonate pathway and the pleiotropic effect they exhibit, leading to the inhibition of proliferation, migration, invasion, survival, and stemness. These complex mechanisms have attracted significant attention [34,35] and their concise descriptions will be presented in the following section (Section 4. Basic understanding of the molecular mechanism of anti-cancer properties of statins).

## 3. Statins in Primary and Secondary Prevention of Cardiovascular Diseases

Low-density lipoprotein (LDL) cholesterol and increased total cholesterol (TC) levels are both risk factors for cardiovascular diseases (CVDs) [36]. Statins, by their primary mode of pharmacological action, are effective at reducing lipid blood levels; thus, they also lower the risk for CVD [37].

In primary prevention, statins are used to lower the risk of developing CVDs in patients without disease symptoms. This includes individuals who have risk factors for CVDs, such as high blood pressure (BP), hypercholesterolemia, obesity, or a family history of heart disease. The use of statins in primary prevention has been the subject of many studies [38,39,40], and the evidence suggests that they can effectively reduce the risk of myocardial infarction (MI), ischemic stroke, and other cardiovascular events. However, the benefits may depend on the individual’s level of risk, and it is important to weigh the potential benefits and risks of treatment on a case-by-case basis.

A systematic review and meta-analysis designed by Cai et al. concluded that the risk of adverse events attributed to statins was low (muscle symptoms, liver dysfunction, or kidney injury) and did not outweigh their effectiveness in avoiding CVDs, indicating that statins generally have a favorable benefit-to-harm ratio [41]. Findings from another study summarized that statin treatment is just one method for preventing CVD in elderly adults. Treatment for hypercholesterolemia should begin much earlier than 75 to 80 years, and statin therapy should be stopped when palliative care is initiated [42].

Updated evidence assessment and recommendation were carried out in June 2022 by the US Preventive Services Task Force (USPSTF); it showed that statin use has at least a decent net benefit for patients from age 40 to 75 who do not have a previous history of CVDs, which is accompanied by at least one CVD risk factor (such as smoking, dyslipidemia, hypertension, or diabetes mellitus) and a 10-year estimated CVD event risk of 10% or higher [43]. Even though the American College of Cardiology (ACC), American Heart Association (AHA), and USPSTF all recommend statins for the primary prevention of cardiovascular events in individuals with a major risk (most frequently defined as 7.5% risk of major adverse cardiovascular events, MACE, within ten years), the size of the effect has yet to be reliably determined. A survival meta-analysis found that statins reduced all-cause mortality when administered for primary prevention, and it would take 2.5 years of treatment for patients aged 50 to 75 to prevent just one MACE [44].

In addition, statins are effective in preventing atrial fibrillation (AF). According to research by Hung et al., statins decreased the risk of AF by 28% in Taiwanese patients older than 50 years (adjusted hazard ratio [HR]: 0.72; 95% confidence interval [CI]: 0.68–0.77) [45]. The Danish trial (565,044 individuals) highlighted the duration of statin treatment and showed similar positive effects, with a decreased incidence of AF found in patients on long-term statin therapy [46].

In secondary prevention, statins reduce the risk of further cardiovascular events in individuals who have already had a myocardial infarction, an ischemic stroke, or other cardiovascular events. The proof for using statins is very strong in secondary prevention, and they are widely recommended as a standard treatment option [47].

A retrospective study in collaboration between four US healthcare systems was conducted by Tescon et al. The overall risk reduction in MACE was found to be 18% (HR 0.82, 95% CI: 0.70 to 0.95, *p* = 0.007) and was more crucial in the first 180 days (HR 0.72, 95% CI: 0.60 to 0.86, *p* < 0.001). The reduction in the nonfatal MACE number was nonsignificant, 19% (rate ratio 0.81, 95% CI: 0.49 to 1.32, *p* = 0.394), and the reduction in the risk of all-cause death was 65% (HR 0.35, 95% CI: 0.22 to 0.56, *p* < 0.001). The main advantage of taking statins was protection against premature death [48].

Contrary to this, the findings from a retrospective cohort study from 2009 to 2017, carried out by Thalmann et al., concluded that statin usage is still unsatisfactory for the secondary prevention of CVDs, particularly in elderly patients, women, and those who have had ischemic stroke, myocardial infarction, or peripheral arterial disease (PAD) hospitalizations [49]. A meta-analysis study discovered that statins for secondary prevention in patients with ischemic stroke or transient ischemic attack (TIA) do not appear to alter all outcomes related to stroke and all causes of mortality; however, they do reduce the relative risk of recurrent ischemic strokes by nearly 20% and the risk of cardiovascular events by more than 20% [50].

Another retrospective study aimed to compare adherence and discontinuation of statins in lowering the incidence of an initial CVD in high-risk people (primary prevention) and subsequent CVD events (secondary prevention). In comparison to the primary prevention group, the secondary prevention group was 1.55 (95% CI: 1.51–1.59) times more adherent and 0.67 (95% CI: 0.65–0.69) times more likely to discontinue the therapy [51].

The association between the incidence of recurrent venous thromboembolism (VTE) and statin use was the conclusion of a meta-analysis enrolling 8.6 million participants. In primary prevention, statin use appeared to have a protective effect on VTE (RR 0.78, 95% CI: 0.72–0.85), whereas, in secondary prevention, it was also related to a 26% reduced risk of recurrent VTE (RR 0.74, 95% CI: 0.70–0.78) [52].

Overall, statins are an important tool in preventing and managing CVDs.

## 4. Basic Understanding of the Molecular Mechanism of the Anti-Cancer Properties of Statins

While statin use can effectively mitigate CVDs, studies have also shown that this group of drugs possesses anti-cancer properties. The following subsection presents an insight into the molecular mechanisms of statins’ anti-cancer activity and introduces a clinical perspective on statins’ role in liver cancer.

The anti-cancer mechanisms of statins are fundamentally rooted in their lipid-lowering activity and their ability to interfere with the mevalonate pathway. The indispensable role of statins in interrupting the mevalonate pathway-cancer cell axis is vital for their potential clinical benefit as a ground-breaking therapy that supports standard treatments for liver and biliary tract cancers.

The mevalonate pathway is a crucial signaling pathway essential for the synthesis of isoprenoids, such as cholesterol, vitamin D, and lipoproteins. It plays a significant role in tumorigenesis, with increased cellular demand and depletion of mevalonate and its intermediates being strongly linked to the presence of carcinogenic lesions [53]. These, in turn, optimize the availability of said metabolites and encourage cancer cell adaptability [54], allowing for further tumor cell synthesis and population growth in the body to occur [55].

Statins’ competitive inhibition of HMGCR and subsequent suppression of the mevalonate pathway are readily realized, notably in routine hyperlipidemia treatment. Not only do statins prove to be essential in lipid profile management, but they simultaneously cease processes that encourage tumorigenesis. Cancer makes use of the body’s LDL-C stores and utilizes them towards tumor cell membrane formation [56,57]. Statin use has the ability to simultaneously increase LDL receptor (LDLR) expression in the liver while inhibiting cholesterol production [58]. This enables serum low-density lipoprotein cholesterol (LDL-C) to be removed, proving LDL-C elimination to be beneficial for tumorigenesis inhibition and cancer progression.

Statins’ anti-cancer properties are not only limited to increased LDL receptor expression but also aid in the restriction of non-cholesterol metabolite production. Mevalonate pathway products, such as isopentenyl pyrophosphate (IPP), farnesyl pyrophosphate (FPP), and geranylgeranyl pyrophosphate (GPP), all enable protein prenylation of GTPases, mainly Ras and RhoA proteins [59]. Limitations on these processes impede the alteration of various cancer pathways, thereby increasing apoptosis while decreasing angiogenesis, inflammation, and metastasis [60]. All of which are crucial in the fight against cancer development and progression.

The functional effects of statins are not confined solely to the modulation of the mevalonate pathway; they have also shown a range of anti-cancer properties. A review of the available literature shows that statins may inhibit angiogenesis and intensify apoptosis and autophagy in cancer cells. By modulating the tumor microenvironment (TME), statins can effectively prevent cancer progression. Although these studies primarily focus on cell lines [55] rather than liver or biliary tract cancers, they provide a fascinating insight into the molecular aspects of the anti-cancer effects of this group of drugs and serve as a basis for the clinical and potential use of statins in oncology.

Angiogenesis promotes the formation of new blood vessels from pre-existing vessels, playing a critical role in cancer progression [61]. The influence of statins on angiogenesis lies in their impact on Rho, vascular endothelial growth factor (VEGF), and endothelial cell migration. It demonstrates that statins are anti-angiogenic. This anti-angiogenic outcome of statins in cholesterol-independent patients is accomplished by inhibiting the RhoA/focal adhesion kinase/AKT pathways [60]. The initial development of tumor blood vessels, relying on endothelial progenitor cells through vasculogenesis, could be disrupted by statins, potentially impeding tumor growth. Furthermore, statins possess the capability to hinder blood flow to the liver, thus limiting the growth of tumors [62].

Statins can induce apoptosis in cultured cancer cells by inhibiting the production of geranylgeranyl pyrophosphate (GGPP), consequently decreasing levels of phosphorylated ERK1/2 and Akt [63,64]. Statins’ role in GGPP suppression hinders the proteasome pathway along with FPP degradation caused by their inactivation [65]. This enables statins to exert their inhibitory effects on cell growth in cancer cells. Their anti-cancer properties include having an inhibitory effect on DNA methyltransferases (DN-MTs) activity, controlling demethylation and activation of BMP signaling transits with stem-like properties in cancer cells, and promoting p21 cell cycle arrest. Thus, it enhances the growth inhibition of p21 and p27 by suppressing degradation via the proteasome pathway [66,67]. In CCAs, apoptosis disturbs the colocalization of Rac/Lipid rafts, depressing Rac1 activity [68], and terminating the expression of ATP-binding cassettes (ABCA1 and ABCG1) [69]. All of these actions demonstrate statins’ inhibitory effect on CCA cell survivability and subsequent pathway interruption.

Simultaneously, statins can prevent HCC by suppressing HCV replication. Recent studies have shown an intriguing association between the use of statins and a decreased risk of HCC. This observation shows us the potential use of statins to reduce the risk of HCC development in patients infected with this virus [65].

GGPP and FPP are known as the final products of the mevalonate pathway and play a vital role in the proliferation of malignant cells [65]. Their absence leads to cell death via activation of the Ras and Rho pathways. The role of Ras is to regulate cell proliferation and differentiation, whereas Rho controls actin in the cytoskeleton, gene expression, and proliferation. Research demonstrates that statins induce apoptosis by inhibiting Ras signaling pathways in human hematopoietic tumor cells. Additionally, it has been observed that statins inhibit the activation of certain Rho proteins and reverse the metastatic phenotype of human melanoma cells in vitro [70,71,72]. Furthermore, statins can express a proapoptotic effect through an HMG-CoA reductase-dependent mechanism that activates caspases and decreases Bcl-2 [73]. This occurs through the modulation of the mitogen-activated protein kinase 1 (MAPK)/extracellular signal-regulated kinase (ERK) pathway. As a result, statins inhibit signaling activation, allowing for the induction of molecular targets in HCC treatment [74,75,76].

The role of statins cannot only be limited to the aforementioned mechanisms; they, too, have an affinity for AMP-activated protein kinase (AMPK) pathway activation, demonstrating yet another mechanism by which they can impede cancer development. AMPK is a cellular energy sensor that inhibits cell proliferation and induces apoptosis in cancer cells. Furthermore, statin-associated activation of AMPK leads to reduced lipid accumulation in the liver, which may diminish the risk of liver cancer. It can help guide us to the role and action of autophagy in tumor cell apoptosis [77].

Autophagy is crucial when referring to the progression of liver cancer. Its importance can be best shown via its regulatory role in numerous signaling pathways like PI3K-AKT-mTOR, AMPK-mTOR, EGF, MAPK, Wnt/β-catenin, p53, and NF-κB pathways. They can be implicated in the regulation of statin-mediated autophagy associated with the mevalonate pathway. Statins have the capacity to trigger autophagy via the AMPK-mTOR signaling pathway, primarily by consuming geranylgeranyl pyrophosphate (GGPP), thus initiating autophagic reactions. The induced activation of autophagy by statins might exert a significant and robust influence, diminishing their anti-cancer efficacy. This process is under the regulation of two signaling pathways: the mammalian target of rapamycin (mTOR) and AMPK [78].

In cancer, autophagy plays two roles. Initially, it suppresses tumor development by inhibiting inflammation, promoting genomic stability, and eliminating oncogenic proteins. Conversely, autophagy can aid tumorigenesis by providing nutrients and energy and promoting angiogenesis [79].

Considering the dual role of autophagy in hepatocellular carcinoma (HCC), the importance of sustaining balanced autophagy activity within the PI3K/AKT/mTOR pathway to enable statins to stimulate autophagy in HCC. This action triggers an autophagic response mechanism due to the use of statins on the PI3K/AKT/mTOR signaling pathway [78].

Through the activation of the AMPK/p21-dependent endoplasmic reticulum stress response, statins inhibit cell growth by inducing apoptosis and simultaneously promote cell survival via the induction of autophagy. Thus, demonstrating autophagy’s dominant and selective nature and its crucial involvement in cellular processes that enable it to play a primary role in hepatocarcinogenesis.

Cancer progression is strongly associated with metabolic reprogramming and hypoxia [80]. The tumor’s microenvironment (TME) pertains to a tumor’s composition, the molecules it synthesizes and releases, and can determine whether the cancer can continue to thrive. Dynamic changes and mutual interactions between cancer cells and the TME [81] constitute a fundamental role in tumor initiation, proliferation, and reaction during treatment [31]. In response to a sudden limitation of lipids and/or oxygen, transcription factors such as SREBP2 or RORγ [82], as well as other mevalonate pathway enzymes, trigger increased upregulation in the tumor [83], allowing increased cholesterol synthesis and uptake in cancer cells. This emphasizes the importance of statins’ inhibitory function on the mevalonate pathway, thus enabling adequate regulation of metabolic TME and cancer progression.

**Figure 2:**
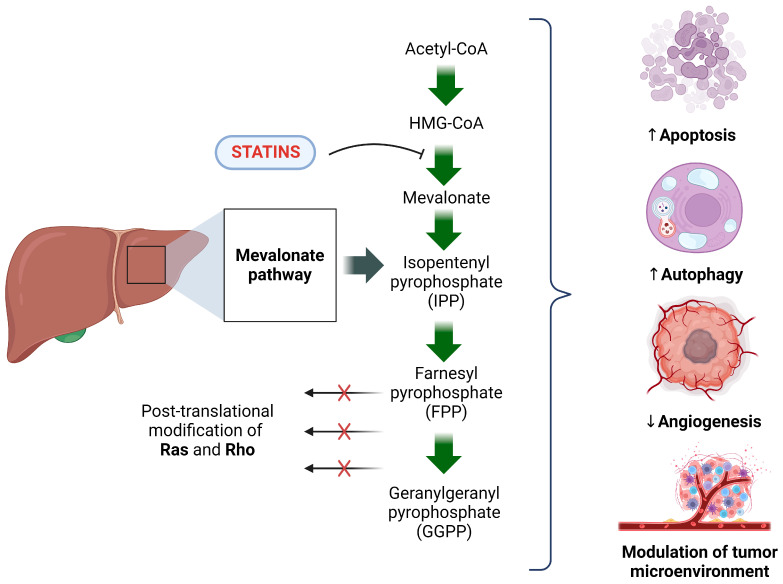
The molecular processes underlying statin anti-cancer effects in liver cancer. The use of statins in mevalonate pathway modulation allows for the inhibition of tumorigenesis. Statins competitively bind to HMGCR, an enzyme responsible for the catalysis of HMG-CoA to mevalonate, consequently reducing the amount of farnesyl pyrophosphate (FPP) and geranylgeranyl pyrophosphate (GGPP). Statins’ disruption of the mevalonate pathway impacts the prenylation of Ras and Rho proteins. These impede the potential development and progression of cancer cells. Statin-induced molecular changes lead to an increase in apoptosis and autophagy within cancer cells. Thus, inhibiting angiogenesis while also modulating the tumor microenvironment (TME) effectively impeding cancer cell growth. (Created with BioRender.com, accessed on 15 October 2023).

It is essential to highlight that the molecular processes underlying statin anti-cancer effects in liver cancer may differ depending on the specific subtype of liver cancer and the cellular environment. Therefore, further research is needed to understand these pathways and their therapeutic implications for treating liver cancer. Nevertheless, by altering the mevalonate pathway, apoptosis, and angiogenesis, statins may modulate various stages of carcinogenesis. A visual representation of these processes can be found in Figure 2. Statins’ mechanisms seem intriguing, considering that some of them may have clinical significance, exceeding the primary importance of statins as anti-cholesterol drugs and shifting the importance of this group of drugs towards oncology.

## 5. Statins in HCC Prevention

Hepatocellular carcinoma (HCC) accounts for approximately 90% of primary liver cancers, is among the top five cancer-related deaths worldwide, and is predicted to have a rise in the number of cases of 55% by the year 2040 [84]. Consequently, it is only justified to address the risk factors for this type of cancer in a large-scale effort to decrease its global burden; to this point, numerous researchers have focused on preventive actions for decreasing the incidence of HCC. As far as drug repurposing goes, statins have attracted much attention in the past two decades for their anti-cancer proprieties and potential role in HCC prevention, in several clinical and epidemiological studies [85,86,87,88], on top of their conventional use as lipid-lowering drugs. These correlations were made due to the higher incidence and mortality of HCC observed in patients with features of metabolic syndrome [89]. The enthusiasm for statin use is nevertheless tempered by their dose-dependent risk of hepatic injury, with a prevailing cholestatic pattern, rendering them far less used for patients with liver disease [90].

Statins exert their effect by competitively inhibiting 3-hydroxy-3-methyl-glutaryl-CoA (HMG-CoA) reductase (HMGCR); the primary outcome is represented by the reduction in de novo cholesterol synthesis and the decrease in low-density lipoprotein (LDL) receptor expression [91]. Their anti-tumoral effect seems to be related to signaling cascades that are likewise associated with HCC development; it was revealed that statins have the ability to downregulate specific signaling pathways [92], thus promoting cell apoptosis; they also limit the degradation of kinase inhibitors, leading to the prevention of tumor development [93] or employ direct anti-fibrotic effects in some HCC populations [86]. Furthermore, lower cholesterol levels have independently been associated with the risk of liver cancer [31].

Numerous clinical studies were conducted on this topic (Table 1), and while one of the first retrospective cohort studies using the Danish National Health Service database showed no significant association between statin use and risk of any cancer, including liver cancer (HR 1.16, 95% CI: 0.46–2.90) [94], subsequent cohort studies employing large databases such as the UK’s Clinical Practice Research Datalink or the Korean National Health Insurance consistently reported a chemopreventive role for statins in HCC for the general population, regardless of prior liver disease [95,96]. Concerning populations at risk for HCC development, several retrospective cohort studies were conducted, mainly assessing patients infected with HBV or HCV. Two studies conducted on Asian HBV-infected patients treated with nucleoside/nucleotide analogues revealed the protective role of statins [97,98]. Similar results were reported in HCV-infected patients, with a dose-dependent reduction in HCC risk: adjusted HRs of 0.66, 0.47, and 0.33 for statin use of 28 to 89, 90 to 180, and >180 high cumulative defined daily dose per year, respectively [86]. Whereas the NAFLD-related HCC population prompts higher statin use, there are fewer studies regarding this category of patients; still, a retrospective cohort study including 1072 patients with NASH-related fibrosis (F3 and cirrhosis) receiving statin treatment reported a protective effect against HCC in a dose-dependent manner for NASH-related cirrhosis patients (HR, 0.40; 95% CI: 0.24–0.67) [99].

A meta-analysis of 20 studies and a total of 2,668,497 patients [100] evaluated the beneficial role of statins for HCC prevention (OR: 0.573; 95% CI: 0.491–0.668, I2 = 86.57%); the study included six cohort studies, four of which found a positive association between statin use and a significantly lower risk of HCC development. Eleven case-control studies have also established that statin use is beneficial in reducing the risk of HCC. Another meta-analysis that included ten studies similarly promoted the use of statins for successful HCC prevention (adjusted OR: 0.63; 95% CI: 0.52–0.76), although their results were heterogeneous partly due to the predominantly Asian population [87]. Additionally, a more recent meta-analysis comprising 32 studies and a population of 4,963,518 patients revealed a significant 42% reduction in HCC incidence, determining that statin users are less likely to develop HCC than non-users (adjusted OR: 0.58; 95% CI: 0.51–0.67). The study uncovered that statins play a protective role in both chronic liver disease patients (OR, 0.52; 95% CI: 0.40–0.68) and the general population (OR, 0.60; 95% CI: 0.50–0.72); the authors also called attention to the fact that the chemopreventive effects of statins are more prominent in Asian populations, pointing out the need for randomized clinical trials in Asian and Western populations to establish the benefit of statin use based on liver cancer etiology [101]. Seeing that metformin and aspirin use was also associated with lower HCC incidence, a meta-analysis of ten studies (n = 1,774,476) examined the connection between statins, aspirin, or/and metformin use and HCC risk; they concluded that only statin use was linked with overall HCC risk reduction (HR: 0.52; 95% CI: 0.37–0.72) in all subgroup analyses that accounted for concurrent medications [102]. In a similar context, a nationwide nested control study conducted by Kim et al. [103] explored the relationship between statin use in patients with incident type 2 diabetes mellitus and the risk of developing HCC, revealing a significant risk reduction in a subset of patients with liver disease (aOR = 0.27, 95% CI: 0.14–0.50), but not significant in patients without liver disease. In an effort to ascertain the causative relationship between statin use and HCC chemoprevention, there are currently two ongoing clinical trials: a phase IV prospective randomized clinical trial that includes patients with early-stage HCC (BCLC 0 and A) after curative treatment (ablation or resection) that is evaluating the role of atorvastatin in the reduction in HCC recurrence over a period of three years [104] and a phase II trial that is assessing the potential role of simvastatin in preventing liver cancer in patients with liver cirrhosis [105]. Hopefully, these trials will have a positive significance for at-risk liver cancer patients.

It was postulated that the reduction in HCC development is directly proportional to the statin dosage. A connection between a high cumulative defined daily dose (cDDD) (as recommended by the World Health Organization for comparison of medications) and the risk of developing liver cancer was suggested. A high >365 cDDD was necessary to diminish the risk of liver cancer significantly, according to Chen et al. [106] in a population-based cohort study on HBV-infected patients who were prescribed both statins and metformin; Kim et al. also found a dose-dependent risk reduction, with doses greater than 720 cDDD proving the most effective [107]. The type of statin used for HCC prevention was likewise studied: the theory that atorvastatin, simvastatin, and fluvastatin are lipophilic statins, therefore more liver-specific, has been hypothesized; a meta-analysis revealed that simvastatin (OR, 0.53, 95% CI: 0.48–0.59), atorvastatin (OR, 0.54, 95% CI: 0.45–0.64), or rosuvastatin (OR, 0.55, 95% CI: 0.37–0.83) had significantly reduced HCC incidence compared to hydrophilic statins (OR, 0.77, 95% CI: 0.58–1.02) [101]. A further prospective cohort study using the Nationwide Swedish Registry, including HBV and HCV-infected patients, reported a significant reduction in HCC incidence with lipophilic statin use (HR, 0.56; 95% CI, 0.41–0.79) [108]. However, other studies showed no statistical differences between the type of statin used and its preventive role [96,109,110].

Limitations to most existing studies are represented by failure to control other confounders, such as risk factors for HCC, like HCV, HVB, alcoholic liver disease, NASH, or concomitant medications, selection bias, exposure misclassification, or measurement bias. A substantial number of patients with HCC also present with type 2 diabetes mellitus and take metformin, another drug associated with liver cancer prevention. Moreover, since higher cholesterol levels have been negatively associated with the risk of hepatocellular carcinoma, a study conducted by Yin et al. revealed that after adjusting for cholesterol levels, the chemopreventive effect of statins disappeared (HR = 1.16, 95% CI: 0.80–1.69), suggesting that only high baseline cholesterol levels are associated with reduced HCC risk, not statin use per se [111]. Eliminating confounding data in the individual studies presented is impossible. Thus, their results must be cautiously interpreted. Furthermore, data are lacking regarding the dosage and duration of statin therapy in many studies. Overall, while it is advisable to take heed of all the limitations presented, most studies revealed a beneficial effect of statin use. In addition to being currently underutilized, and while prospective RCTs are pending, they may be taken into consideration for patients with liver disease who are at risk for HCC development.

**Table 1:** Clinical studies and trials on statin use for prevention of hepatocellular carcinoma.

Study	Design	Population	Follow-Up (Years)	Findings
Friis et al. [94]	Retrospective cohort study 1998–2002	n = 348,262	3.3	No beneficial effect for statin use (HR 1.16; 95% CI: 0.46–2.90)
McGlynn et al. [73]	Case control study 1988–2011	n = 5835	-	Significant HCC risk reduction for patients with liver disease and diabetes (aOR, 0.55; 95% CI: 0.45–0.69)
Tran et al. [96]	Case-control study 2000–2011	n = 9852	2	Significant HCC risk reduction (aOR, 0.44 (95% CI: 0.33–0.58)
Kim et al. [103]	Case-control study 2002–2013	n = 1374	12	Significant HCC risk reduction in patients with liver disease and incident TD2M (aOR = 0.27, 95% CI: 0.14–0.50)
Goh et al. [97]	Retrospective cohort study 2008–2012	n = 7713 HBV-infected patients	7.8	Statin use is associated with lower risk of HCC (aHR 0.36, 95% CI: 0.19–0.68)
Hsiang et al. [98]	Retrospective cohort study	n = 53,513 HBV-infected patients	4.6	Statin users had a 32% risk reduction for HCC (wSHR 0.68; 95% CI: 0.48–0.97)
Tsan et al. [86]	Retrospective cohort study 1999–2010	n = 260,864 HCV-infected patients	10.7	Reduction in HCC risk in a dose-dependent manner: aHRs, 0.66, 0.47 and 0.33 for statin use of 28 to 89, 90 to 180, and >180 cDDDs per year
Pinyopornpanish et al. [99]	Retrospective cohort study 2002–2016	n = 1072 NASH-related liver fibrosis (F3 or cirrhosis)	4.6	Statin use associated with lower risk of HCC (HR, 0.40; 95% CI: 0.24–0.67)
Khazaaleh S et al. [100]	Meta-analysis	n = 2,668,497	-	Significant HCC reduction in statin users vs. non-users (OR 0.573; 95% CI: 0.491–0.668, I2 = 86.57%)
Wang et al. [101]	Meta-analysis	n = 4,963,518	-	Significant HCC reduction in statin users vs. non-users (aOR, 0.58; 95% CI: 0.51–0.67)
Singh S et al. [87]	Meta-analysis	n = 1,459,417	-	Statin users were less likely to develop HCC than statin non-users (aOR, 0.63; 95% CI: 0.52–0.76)
Zeng R et al. [102]	Meta-analysis	n = 1,774,476	-	Statin use was associated with HCC risk reduction in all subgroup analysis (HR: 0.52; 95% CI: 0.37–0.72)
NCT02968810, United States [105]	Randomized prospective clinical trial	Patients with liver cirrhosis	-	Simvastatin for preventing liver cancer development in liver cirrhosis patients
NCT03024684, Taiwan [104]	Randomized prospective clinical trial	Patients with HCC BCLC 0, A	-	Atorvastatin for prevention of HCC recurrence after curative treatment

Abbreviations: HR—hazard ratio; OR—odds ratios; aHR—adjusted hazard ratio; aOR—adjusted odds ratio; CI—confidence interval; HCC—hepatocellular carcinoma; cDDD—cumulative defined daily dose; TD2M—type 2 diabetes mellitus; NASH—non-alcoholic steatohepatitis.

## 6. Statins in HCC Treatment

The emergent role of statins in the treatment of chronic liver disease is a current hot topic in hepatology and has been widely documented, alongside the revamped interest in the role of metabolic pathways in halting liver disease progression and preventing decompensating events [112]. The recent evidence regarding the beneficial role of statins in reducing portal hypertension and improving overall survival in patients with advanced liver disease has even garnered mainstream validation, being formalized as a recommendation in the most recent Baveno VII position paper [113]. However, while there are numerous reports on the potential beneficial role of statins in treating hepatocellular carcinoma (HCC), the evidence is less robust. Hence, all the available results should be taken with a grain of salt, considering the quality of the studies, the potential sources of bias, and the chances of bench-to-bedside translation.

Basic science protocols on cell lines and animal models have unequivocally pointed towards the therapeutic effect of statins in HCC. A pioneering study published by Sutter A. et al. in 2005 reported that statins inhibited the proliferation of HCC cells in two distinct cell lines by inducing apoptosis and blocking the cell cycle in the G1/S stage [114]. Congruent evidence regarding the proapoptotic benefits of simvastatin alone [115] or in combination with a selective cyclooxygenase-2 inhibitor (NS398) has reinforced the premise [116]. Similar results were also obtained in animal models, as simvastatin-induced apoptosis and suppressed HCC in rats [117]. While the previously discussed studies analyzed statins as an independent therapeutic method, there have also been reports on statins as adjuvant co-drugs, aiming to improve or preserve HCC sensitivity to established regimens, such as sorafenib or anti-programmed death ligand-1 (PDL-1). One of the pathways to sorafenib resistance is intratumoral hypoxia [118], and there is experimental evidence that using statins + sorafenib can help circumvent this pathway [119]. Other pathways to sorafenib resistance, such as enhanced oxidative phosphorylation and aerobic glycolysis, also appear to benefit from the addition of statins, leading to a temporary increase in apoptosis and sorafenib re-sensitization [120]. Furthermore, there is evidence that statins might generate substantial tumor microenvironment alterations, which might bear prognostic significance, especially concerning immunotherapy treatment response [121,122], with one report suggesting that atorvastatin attenuates PDL-1 induction in HCC cells, hinting towards a potential role of combination therapies [123].

Yet, however promising, these findings had a sub-par translation to clinical practice. An initial randomized controlled trial published in 2001 reported a significant survival benefit for the pravastatin group in patients with advanced HCC undergoing transarterial chemoembolization (TACE) followed by a two-month course of 5-fluorouracil (median survival 18 vs. 9 months, *p* = 0.006) [124], reinforced by another similar study published by Graf H et al. in 2008 [125]. However, these results were not reproduced by the larger-scale French PRODIGE-11 multicentric trial, which reported no benefits from the addition of pravastatin to simvastatin in patients with advanced HCC [126]. Another phase II trial, which compared sorafenib, pravastatin, sorafenib + pravastatin, and best supportive care in patients with Child–Pugh B cirrhosis and HCC, found no positive effect of statins [127]. The role of adding statins in palliative HCC care has also been investigated [128], and data from a large-scale populational study suggested that statin-based palliative regimens improve HCC-related mortality [129]. However, this study has significant limitations. Although it included approximately 20,000 subjects, of which almost 10% used statins, the data were retrospectively collected through electronic database records, raising significant selection bias issues [129]. Therefore, the data might be skewed towards the “healthier” subjects (less advanced liver disease, better performance status, more compliant patients) receiving statins, as these represent some of the most common flaws in large pharmacoepidemiologic studies [130].

On the other hand, there appears to be robust evidence regarding the role of statins in patients with HCC amendable for curative intent treatment, such as surgery, liver transplantation, or loco-regional therapies. Studies that included patients undergoing liver resection for very early and early HCC (BCLC-0 and A) have reported improved outcomes for patients under statin therapy. A Japanese study published in 2018, which included 643 patients, of which 6.7% were under statin therapy, reported a significant improvement in recurrence-free survival (HR 0.42, 95% CI: 0.25–0.71, *p* = 0.001), yet with no significant improvements in overall survival (HR 0.62, 95% CI: 0.30–1.27, *p* = 0.19) [131]. Another study, published by a group from Taiwan with a similar sample size and design, reached a similar conclusion, as the patients in the statin group had a lower risk of recurrence (HR = 0.354, *p* < 0.001) without an increase in overall survival [132]. A recently published meta-analysis by Khajeh E et al. confirms these reports, as statin use was associated with lower recurrence up to 5 years after surgery (OR 0.28, 95% CI: 0.19–0.42, *p* < 0.001) [133]. Regarding loco-regional therapies, such as radiofrequency ablation or percutaneous ethanol injection, a large-scale retrospective study suggested that statin use might improve overall survival [134]. However, this result was only apparent in specific subgroup analyses. The study is at risk for the same selection biases discussed above, given that the data were retrospectively collected from electronic insurance database records. The strongest evidence comes from studies on liver transplantation for HCC. Two recently published studies with similar designs, which both included over 400 patients undergoing liver transplantation, reported significant outcome improvements in patients using statins. In both studies, patients in the statin groups had significantly lower recurrence rates [135,136]. Moreover, in the study published by Lee H et al., patients in the statin group also had lower all-cause (HR 0.3, 95% CI: 0.2–0.5, *p* < 0.001) and HCC-related mortality (HR 0.4, 95% CI: 0.2–0.9, *p* = 0.03) [136].

When putting these findings into context, one can notice that statins maintain a preventive effect (for HCC recurrence) instead of having an independent therapeutic impact, in line with the discussion in the previous chapter. To this date, there appears to be a poor bench-to-bedside translation for systemic therapy in HCC. However, given the relatively low risk along with the proven beneficial effects on the course of the underlying liver disease, future studies might validate what is currently hinted at by laboratory data.

## 7. Statins in CCA Prevention

Different from other cancer entities, all cholangiocarcinoma subtypes have something in common: a late stage of diagnosis and a poor outcome. Therefore, it is easy to understand why one of the best approaches is to try to prevent instead of treat the disease. The risk factors are indeed poorly defined and highly heterogeneous, but disorders of lipid metabolism (and subsequent inflammatory and immune-mediated reactions of the biliary tree) are in part responsible for cholelithiasis, infection with bacteria and parasites, and primary sclerosing cholangitis [137]. Therefore, inhibitors of the hepatic synthesis of cholesterol have been investigated for their ability to prevent cholangiocarcinoma [138].

Considering the rarity of biliary tract cancers, the amount of evidence on the effects of statins on CCA prevention is rather scanty; however, some educated guesses are warranted. To date, nine studies have evaluated the role of statins in CCA prevention. The main finding from each study is depicted in Table 2. Moreover, one meta-analysis published in 2020 that included seven studies and 6,251,187 participants showed that the risk of CCA among individuals who use statins compared with individuals who do not use statins was significantly lower with a pooled odds ratio of 0.68 (95% CI: 0.52–0.89; F—96%) [139]. Not least, another meta-analysis published this year that included eight studies also showed a lower risk for cancer in statin users versus non-users, especially in CCA patients (pooled aRR for iCCA was 0.60; 95% CI: 0.38–0.94) [140]. Overall, the data on a possible favorable effect of statins on CCA risk remain relatively modest. We still have limited information on confounding factors (e.g., hepatitis B and C virus infection, parasite infections) in administrative record linkage datasets; it is not known how many CCA cases were pathologically proven, and there is no data regarding the effects of statins on CCA risk in patients with normal cholesterol levels.

With all this being said, statins might play an important role in specific clinical scenarios, and clinicians need to consider them when trying to provide better care for CCA patients. In subsequent clinical situations, clinicians should also check for cholesterol levels. (a) Patients with bile duct cysts where there is an increased risk for CCA development (odds ratio (OR) = 15.66 (95% CI: 11.58–21.18) for iCCA and an OR = 27.12 (95% CI: 22.06–33.34) for extrahepatic CCA (eCCA) [148] (b) Patients with primary sclerosing cholangitis (PSC) where the risk for CCA is also higher (OR = 21.52 (95% CI: 7.21–26.90) for iCCA and an OR = 40.80 (95% CI: 34.96–47.60) for eCCA [148]; (c) Hepatolithiasis, cholelithiasis and choledocholithiasis—where there is a higher risk for both iCCA and eCCA; the risk seams to increase with gallstone size, epithelium calcification and disease duration [149]; (d) Patients with cirrhosis or viral hepatitis (B and C) are also at risk for iCCA (OR = 8.0, 95% CI: 6.6–9) and eCCA (OR = 3.9, 95% CI: 3.0–5.1) besides the well-known risk of HCC [150]; (e) NAFLD/NASH—an increased risk of both iCCA (OR = 3.52, 95% CI: 2.87–4.32) and eCCA (OR = 2.93, 95% CI: 2.42–3.5) for patients with NAFLD [148]; (f) Parasitic infections (*Opistothorchis viverrini* and *Clonorchis sinensis* infections)—It has been estimated that up to 10% of people chronically infected with these liver flukes will develop CCA, especially iCCA [151], and (g) Environmental exposure to Thorotrast (RR = 300), 1,2-dichloropropane (adjusted RR = 17.1, 95% CI: 3.8–76) and asbestos [152]. The aforementioned clinical scenarios are all associated with a very high or high risk of CCA development. If cholesterol levels are elevated in these situations, the decision to start statin is straightforward. However, if cholesterol levels are normal, the decision is more nuanced. We do not know the number needed to treat to achieve benefit, but it might be within the range that justifies using statins in cancer prevention. Off-label use of statins in these circumstances could be an option while waiting for further investigations.

Several other risk factors for CCA, including inflammatory bowel disease, alcohol, smoking, obesity, type II diabetes, chronic pancreatitis, and duodenal/gastric ulcers, have a strong association with CCA, and statins should be prescribed only if cholesterol levels are elevated.

## 8. Statins in CCA Treatment

Besides the role of statins in CCA prevention, some preclinical and clinical studies (albeit few) have focused on statins in CCA treatment. Regarding preclinical data, some studies performed on human and murine cholangiocarcinoma cell lines demonstrated that statins inhibit cell proliferation, induce apoptosis and autophagy, and increase the expression of tumor necrosis factor-alpha mRNA [153,154]. Furthermore, statins enhance chemotherapeutical agents’ effects [155]. A Japanese team investigated the effects of atorvastatin in combination with gemcitabine in cholangiocarcinoma murine xenograft models with respect to the oncogenic regulation of the transcriptional co-activator Yes-associated protein. Both agents suppressed the proliferation of tumoral cells in vitro in human cholangiocarcinoma cells and induced apoptosis in two cholangiocarcinoma cell lines. Moreover, the individual anti-cancer effect of the two agents was enhanced when used in conjunction, as atorvastatin plus gemcitabine decreased tumor burden in the xenograft model [156].

Another study from Thailand examined the effects of simvastatin with 5-fluorouracil and cisplatin in human cholangiocarcinoma cells, demonstrating many anti-cancer effects in cell lines, the most important being inhibition of cancer cell proliferation and migration. They also demonstrated that simvastatin and atorvastatin increased the effect of the above-mentioned chemotherapeutic agents against cholangiocarcinoma, with simvastatin showing superior enhancement to atorvastatin [157]. Simvastatin also induces increased apoptosis by promoting the production of reactive oxygen species in a dose-dependent manner [158]. An American group from Texas also demonstrated the enhancement of apoptosis by simvastatin by suppressing the activity of the Rac1 protein. Simvastatin reduced the viability of tumor cells in five cholangiocarcinoma cell lines but did not induce apoptosis among cholangiocytes from a normal cell line that served as a control [68]. Lovastatin was also validated as an anti-proliferation-inducing agent in cholangiocarcinoma cell lines. It inhibited the expression of integrin β3, decreased the signaling molecules in the integrin/β-catenin pathway, decreased the function of β-actin within the tumoral cells, and thus restricted cell adhesion [153]. Kamigaki et al. also showed supplementation of growth suppression of cholangiocarcinoma cells when adding statins (pitavastatin and atorvastatin) to gemcitabine, cisplatin, and 5-FU. Both agents induced a significant reduction in tumor cell proliferation by 20.5 and 14.2-fold, respectively, after 96-hour exposures. Cells developed morphological alterations such as decreased size, number, and pseudopod formation [155].

Until now, only two studies have evaluated statins’ “chemotherapeutic” potential in CCA patients. A hospital/clinical-based case-control study evaluating the survival of statin users vs. non-statin users showed that patients with distal CCA who ever used statins had better overall survival. However, this observation was not seen in perihilar CCA patients [146]. Lastly, a recently published study that included 1140 biliary cancer patients evaluated the benefit of concurrent statins with systemic therapy. Patients who received concomitant statins with systemic therapy versus those who received only systemic therapy did not experience improved progression-free (5.5 vs. 5.5 months; hazard ratio (HR) 1.1; *p* = 0.51) or overall survival (12.3 vs. 12.6 months; HR 1.1; *p* = 0.18), respectively [159].

Altogether, whether speaking of prevention or treatment, we do not have data regarding which type of statin is best for clinical practice. Nor do we know if a higher dose or a more extended period of use equals better results. In terms of prevention, as already mentioned, we do have some windows of opportunity, while treating CCA with statins should only be made in clinical trials. Immunotherapy is now part of CCA treatment. Therefore, it would be interesting to study the combined effect of statins and immunotherapy in CCA since, for lung cancer, the combination of statins and immunotherapy was shown to increase progression-free survival and overall survival [160].

## 9. Conclusions and Perspectives

Our primary goal in developing this narrative review was to present statins in a different light than is usually conducted, namely not only as drugs used in hypercholesterolemia but to demonstrate potential therapeutic lines using statins in liver cancers. Our overarching goal has, therefore, been achieved, and the manuscript provides the necessary experimental and clinical data on the role of statins in inhibiting the formation and proliferation of cancer cells. Despite the lack of a clear mechanism to explain this reduction, statin treatment has been linked to a lower risk of developing HCC in patients with chronic liver disease. Numerous preclinical and clinical studies showed that statins can enhance the benefits of commonly used cancer medications when used in combination, despite the lack of clear clinical data supporting the use of statins as monotherapy for cancer. In addition, adjuvant chemotherapy in HCC patients utilizing a regimen that also includes a statin shows promise for increasing survival. Although statin therapy alone or in conjunction with other anti-cancer drugs has produced encouraging outcomes, the precise mechanisms by which they exert their anti-tumor effects frequently remain unknown. Statin potency, physical/chemical characteristics, dosage, and duration of treatment all had a significant impact on the results that were seen. Since they could quickly traverse cell membranes and are effectively absorbed by cancer cells, lipophilic statins like fluvastatin, atorvastatin, simvastatin, and lovastatin were typically favored, even though hydrophilic statins like rosuvastatin and pravastatin are more hepatoselective. Statins are prepared to be studied in carefully planned prospective clinical trials, with lipophilic statins likely leading the way as they have been shown in a significant number of preclinical studies to have anti-cancer properties as well as to be safe and have little to no side effects. Current findings showed a significant relationship between statin users and a lower risk of cancer onset: the lowered risk between rosuvastatin, simvastatin, and atorvastatin and liver cancer reached statistical significance, while the inverse relationship between cerivastatin, pravastatin, and fluvastatin did not. In conclusion, statin drugs are, therefore, valuable support for standard oncological therapies. A severe limitation of their clinical use is the incomplete characterization of their anti-cancer effectiveness in large-scale clinical trials, especially in biliary tract cancers, and potential adverse effects also related to their toxic effect on hepatocytes. Future research should focus on developing new statin molecules with better anti-cancer properties and lower toxicity, e.g., using modern artificial intelligence technologies. It also seems interesting to examine the effect of this group of drugs on metastasis and the potential inhibition of this feature of malignant tumors.

## Figures and Tables

**Table 2:** Studies that evaluated the role of statins in cholangiocarcinoma prevention.

Author and Year	Country/ Region	Study Design	Sample Size		Main Findings
			Statin Users	Statin Non-Users	
Friedman 2008 [141]	USA/ West	Cohort territory-wide healthcare delivery program database	361,859	3,860,801	There is no strong evidence of either the causation or prevention of cancer by statins.
Burr 2014 [142]	UK/ West	Case control 2 territory hospitals	81	275	There were no significant associations between the development of cholangiocarcinoma and statins (OR 0.58; 95% CI: 0.28–1.19)
Peng 2015 [138]	Taiwan/ East	Case control nationwide NIHRD	1560	4788	The overall adjusted OR of statin use-associated CCA was 0.80 (95% CI: 0.71–0.90) and lowered for those with longer medications. A stronger dose-response association was seen when using lovastatin.
Marcano-Bonilla 2022 [143]	Sweden/ West	Population-based cohort nationwide drug registry database	950,635	4,809,847	Statins were associated with a lower risk of BTC (HR, 0.66; 95% CI: 0.56–0.78), iCCA (HR, 0.69; 95% CI: 0.50–0.95), eCCA (HR 0.54; 95% CI: 0.38–0.76), and gallbladder cancer (HR, 0.72; 95% CI: 0.57–0.91)
Liu, Alsaggaf 2019 [144]	UK/ West	Case control nationwide CPRD GOLD database	5544	13,093	Compared with the nonuse of statins, current statin use is associated with a 12% lower risk of BTCs.
Prasai 2019 [145]	USA/ West	Case Control	633	1266	In multivariate analysis, statin use was not associated with a reduced risk of gallbladder carcinoma.
Lavu 2020 [146]	USA/ West	Case control at the Mayo Clinic in Rochester	482	716	Statin use was significantly associated with a 4-fold reduction in the risk of eCCA. The risk reduction was observed among the two subtypes of ECC to varying degrees: 3-fold in pCCA and 16-fold in dCCA.
Tran 2020 [96]	UK/ West	Prospective cohort PCCIU of Scotland, UK biobank of England, Scotland, Wales	395,301	76,550	Statin use was associated with a 39% lower risk of liver cancer in the PCCIU
Chaiteerakij 2013 [147]	USA/ West	Case control Mayo clinic of Rochester	237	969	There is no association between statin use and iCCA risk among patients with hyperlipidemia

Abbreviations: UK = United Kingdom, dCCA = distal cholangiocarcinoma, pCCA = proximal cholangiocarcinoma, eCCA = extrahepatic cholangiocarcinoma, iCCA intrahepatic cholangiocarcinoma, CI = confidence interval, OR = odds ratio, BTC = biliary tract cancer, HR = hazard ratio.
